# Fungal annexins: a mini review

**DOI:** 10.1186/s40064-015-1519-0

**Published:** 2015-11-24

**Authors:** Kamand Khalaj, Elahe Aminollahi, Ali Bordbar, Vahid Khalaj

**Affiliations:** 1 Medical Biotechnology Department, Biotechnology Research Center, Pasteur Institute of Iran, Tehran, Iran; 2 Medicine Faculty, Tehran University of Medical Sciences, Tehran, Iran; 3 Molecular Systematics Laboratory, Parasitology Department, Pasteur Institute of Iran, Tehran, Iran

**Keywords:** Annexin C, Aspergillus, Fungal physiology

## Abstract

The large family of annexins is composed of more than a thousand members which are typically phospholipid-binding proteins. Annexins act in a number of signalling networks and membrane trafficking events which are fundamental to cell physiology. Annexins exert their functions mainly through their calcium-dependent membrane binding abilities; however, some calcium-independent interactions have been documented in the literature. Although mammalian and plant annexins have been well characterized, little is known about this family in fungi. This mini review summarizes the available data on fungal annexins.

## Background

**Fig. 1:**
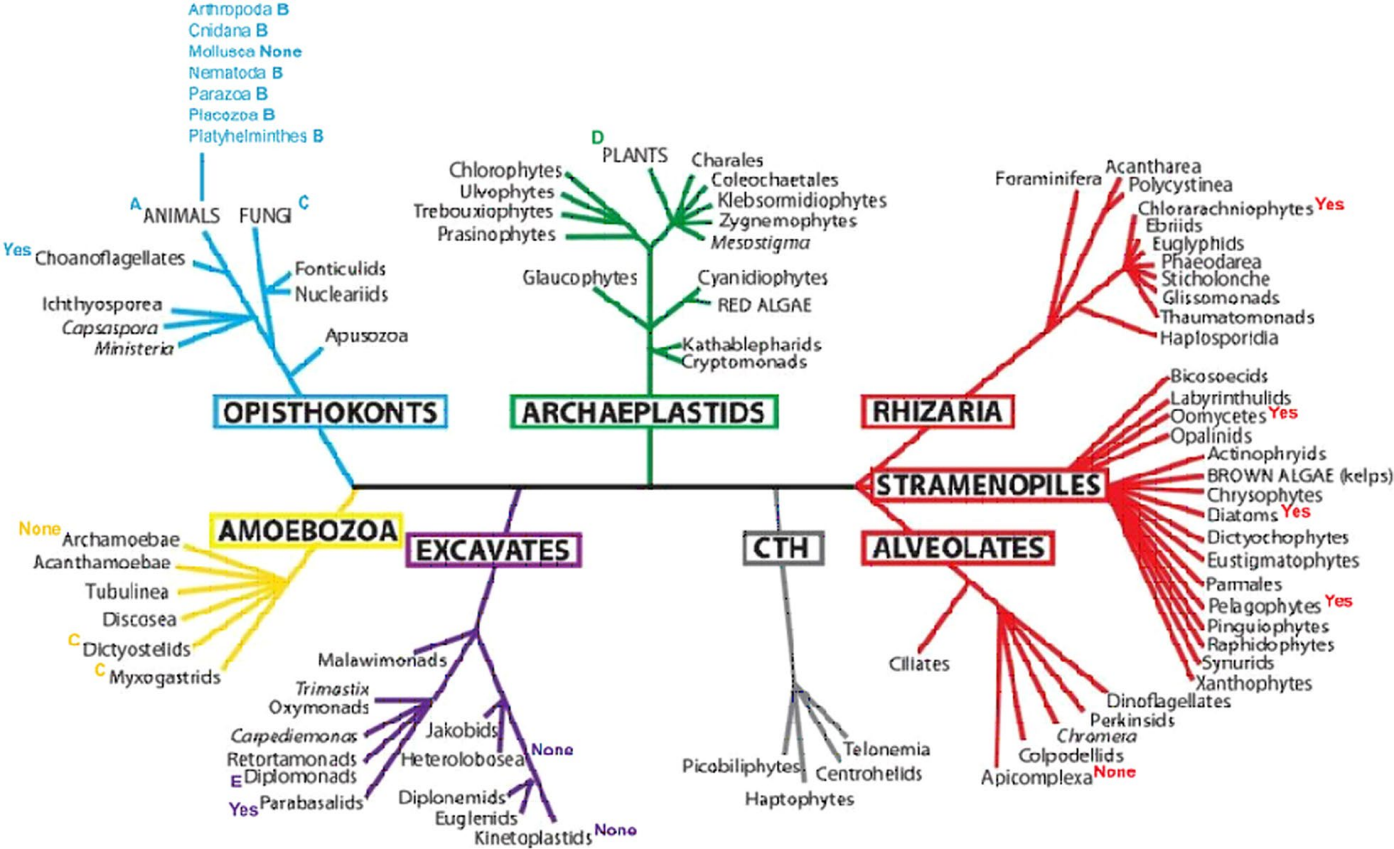
Annexins in *Eukaryota*. The annotation of *A*–*E* indicates the presence of annexins in the groups listed. ‘Yes’ shows the presence of (full-length or partial) uncategorised annexin sequences. ‘None’ indicates the absence of annexins to current knowledge (Cantacessi et al. 2013). Reproduced with permission of Nature Publishing Group. License: 3661771436717

Annexins are a large multi-gene family of calcium-phospholipid binding proteins which are distributed all over the eukaryotic phyla. This super family has been divided into five major families; A family in vertebrates, B family in invertebrates, C family in fungi, mycetozoa and chromalveolates, D family in plants and E family in protists (Moss and Morgan 2004; Morgan et al. 2006) have reported the first single domain bacterial annexin in *Cytophaga hutchinsonii*. However, a recent bioinformatic analysis of bacterial genomes has revealed more members of the annexin family in prokaryotes (F family) (Kodavali et al. 2014). Figure 1 shows the latest scheme and occurrence of eukaryotic annexins (Cantacessi et al. 2013).

The basic structure of annexins is composed of both a conserved core domain and a variable N-terminal region. The core is made up of ~70 amino acid repeats which contain the consensus endonexin sequence GXGT….D/E, and normally participates in the formation of type II Ca^2+^ binding sites (Geisow et al. 1986). Sequences specifying ‘type III’ Ca^2+^ binding sites as well as a phosphatidylserine-binding sequence have been identified (Weng et al. 1993; Montaville et al. 2002). The variable N-terminal region of annexins is supposed to be involved in regulatory functions, including protein–protein interactions and membrane localization (Hayes et al. 2004).

Annexins have been identified in a wide range of fungi including ascomycetes, basidiomycetes and oomycetes (which are taxonomically distinct from true fungi) (Moss and Morgan 2004; Khalaj et al. 2004b). Despite a large number of studies on mammalian and plant annexins, little is known about fungal annexins. In this review, we sought to focus on the available data regarding the fungal annexins.

## History

The first fungal annexin was identified in ascomycete *Neurospora crassa* using a bioinformatic approach (Braun et al. 1998). The identified protein displayed ~35 % identity to its orthologue in slime mold *Dictyostelium discoideum*. In this line, the authors named this gene as anx14 in accordance with *D. discoideum*. Khalaj et al. (2004a) isolated and characterized an annexin homologue from *Aspergillus niger* using a degenerative PCR approach. More members of the fungal annexin family were then identified through EST database searches and some of them characterized experimentally (Khalaj et al. 2004b). Although there is no evidence for the presence of these proteins in yeasts *Saccharomyces cerevisiae*, *Candida albicans* or *Schizosaccharomyces pombe*, the annotation of the *Yarrowia lipolytica* genome has confirmed the presence of an annexin member in this yeast (Khalaj et al. 2004b; Braun et al. 2000; Morgan et al. 2004). In addition, annexins have been found in oomycetes. The first oomycete annexin was isolated from *Saprolegnia monoica* using biochemical methods, followed by DNA sequence analysis (Bouzenzana et al. 2006). A recent examination of detergent-resistant membrane microdomains from *S. monoica* has reconfirmed the presence of different isoforms of a 35-kDa annexin in this oomycete (Briolay et al. 2009). Furthermore, the proteome surveys of *Phytophthora ramorum* and *Phytophthora infestans* cell walls have identified two annexin homologues in these fungal-like organisms (Meijer et al. 2006; Grenville-Briggs et al. 2010).

## Phylogeny

**Fig. 2:**
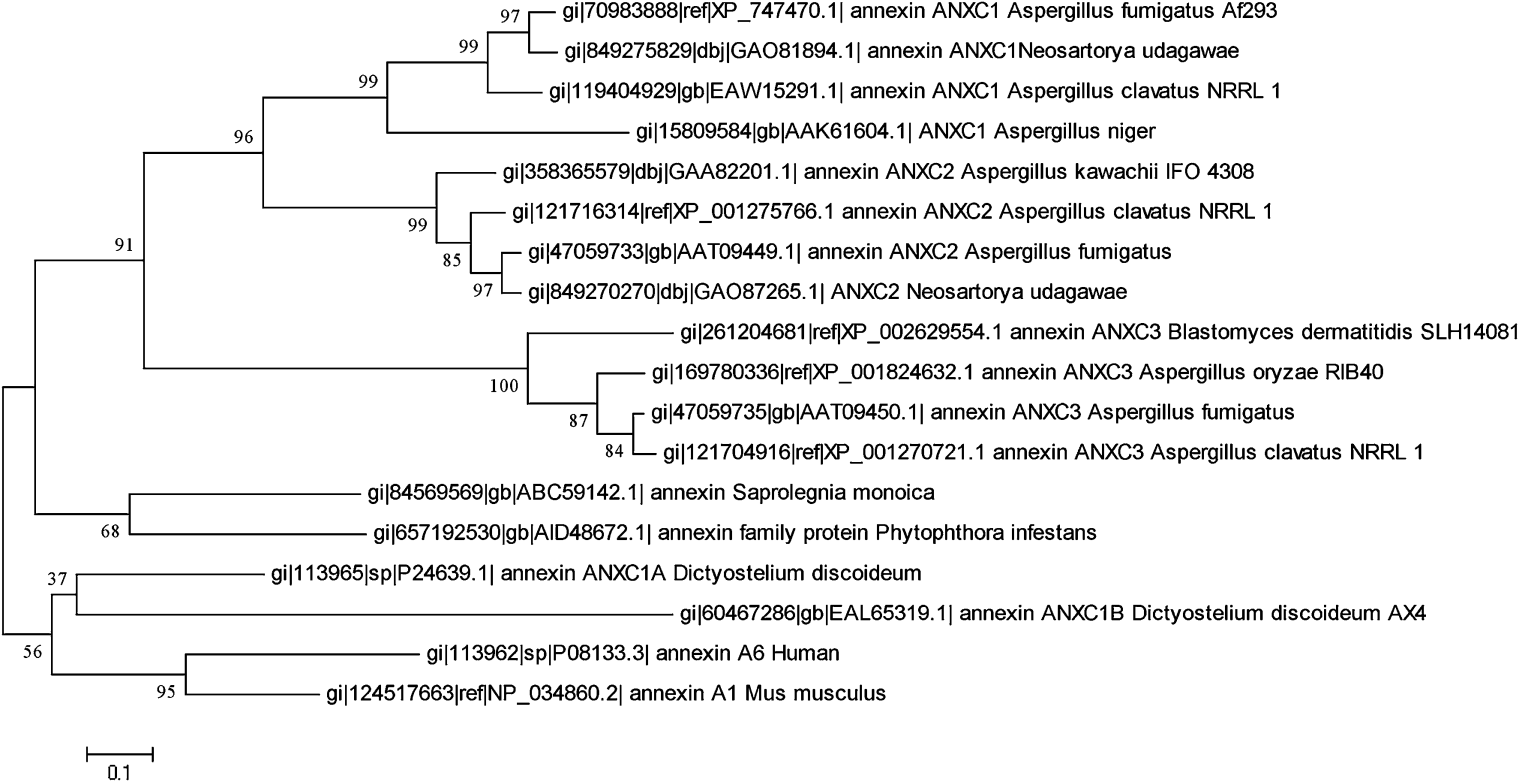
Molecular Phylogenetic analysis of fungal annexins by Maximum Likelihood method. The evolutionary history was inferred by using the maximum likelihood method based on the JTT matrix-based model. The tree with the highest log likelihood (−7071.5193) is shown. The percentage of trees in which the associated taxa clustered together is shown next to the branches. Initial tree(s) for the heuristic search were obtained automatically as follows. A discrete Gamma distribution was used to model evolutionary rate differences among sites [five categories (+G, parameter = 1.2474)]. The tree is drawn to scale, with branch lengths measured in the number of substitutions per site. The analysis involved 18 amino acid sequences. All ambiguous positions were removed for each sequence pair. There were a total of 430 positions in the final dataset. Human and mouse annexins have been included as an outgroup. Evolutionary analyses were conducted in MEGA5 (Tamura et al. 2011)

The phylogeny analysis of annexins has been demanding due to the different number of orthologues and paralogues found in a certain species. Also the accurate phylogeny analysis of annexins requires an extensive and careful examination of all available sequences, gene and protein structures as well as chromosomal locations (Cantacessi et al. 2013). Despite the availability of genome sequence for a large number of fungal genomes, no comprehensive phylogenic analysis of fungal annexins is available. In general, the fungal annexins are poorly clustered with mammalian annexins and grouped in a single clade (ANXC) containing fungal, mycetozoan and chromalveolates annexins (Gerke and Moss 2002; Moss and Morgan 2004). In the present review, we have performed a small scale phylogeny analysis to demonstrate evolutionary relationship between some fungal annexins (Fig. 2). Based on the presented phylogeny tree, ANXC1 and ANXC2 from *D. discoideum* seem to be most closely related to each other than to any other fungal subfamily. These annexins can be considered as distinct subfamilies, but designated as ANXC1A and ANXC1B because of a lineage-specific gene duplication. In a phylogenetic analysis of fungal annexins by Morgan et al. (2004), the Dictyostelium (which is not a fungus) annexin has also been presented as an out group in the fungal annexin phylogeny tree.

In our previous works, we identified three fungal annexins in *Aspergillus fumigatus.* Two of these proteins were called as ANXC3.1 and ANXC3.2 with 40 % identity in the protein level, while the third member, ANXC4, was identified as a novel annexin with significant differences, when compared to other fungal and human annexins (Khalaj et al. 2004a, b). These newly identified proteins were given the name based on next available number in annexin C group (ANXC1 and ANXC2 previously referred to *D. disodium*). The presented phylogeny tree in this review, generally separates ANC3.1, ANX C3.2 and ANXC4 and we make corrections on the previous classification. In this sense ANXC3.1 renamed as ANXC1; ANC3.2 as ANXC2 and ANXC4 as ANXC3. A phylogenetic analysis of the annexin sequence isolated from oomycete *S. monoica* and available EST from other genus of this class, *Phytophthora*, have shown a well grouped oomycete clade with a high bootstrap support (Bouzenzana et al. 2006). The same situation can be observed for oomycete annexins in the presented tree.

## Expression, localization and function

In the higher eukaryotes, depending on the annexin type and the expressing tissue, the gene expression can follow either a constitutive or a highly inducible pattern. In the cellular level, annexins are generally localized in cytosol but partly associated with membranes or cytoskeleton components (Solito et al. 1998; Pratt and Horseman 1998).

Of known members of the ANXC family, two Dictyostelium annexins have been studied in detail (Doring et al. 1991; Greenwood and Tsang 1991; Marko et al. 2006). Based on what Marko et al. (2006) have nicely presented, both Dictyostelium annexins are expressed at a low but constant level during the development. In localization studies, the ANXC1A-GFP fusion protein was present along the cell membrane, in the cytosol, the nucleus and the endosomal compartment, while ANXC1B-GFP fusion labelled the plasma membrane and partially co-localized with Golgi apparatus markers.

A detailed analysis of *anxc1* in filamentous fungus, *A. niger*, confirmed the constitutive expression of this gene (Khalaj et al. 2004a). An ANXC1-GFP fusion construct, driven by an inducible promoter, has been made in our lab and successfully expressed in *A. niger* (unpublished data). Upon the induction of this expression cassette, a cytosolic distribution of the fusion protein has been observed.

In biochemical studies of *Saprolegnia* cell wall components, an annexin homologue has been co-purified with a cell wall enzyme, (1→3)-β-d-glucan synthase. This may demonstrate a membrane localization for this protein (Bouzenzana et al. 2006; Briolay et al. 2009).

The proposed functions of annexins are based mainly on Ca^2+^/phospholipid binding properties in co-operation with various partners (Rescher and Gerke 2004). The most known functions of the annexin family include participation in membrane organization and trafficking events such as endocytosis and exocytosis, ion channel modulation, as well as some extracellular activities like receptor-ligand interactions (Monastyrskaya et al. 2009a, b; Gerke and Moss 2002; Kourie and Wood 2000). However, a number of Ca^2+^ independent activities have also been assigned (Mortimer et al. 2008; Gerke and Moss 2002).

The examination of amino acid sequences of ANXC1 and ANXC2 in *Aspergillus fumigatus* have revealed a well-conserved core region compared to other annexins. The calcium-binding repeat regions I and II were found to be similar to those detected in human annexin VII and ANXC1A in *D. discoideum*. These repeats also contain the consensus phosphatidylserine binding sites. However, for the fourth repeat, there is a very low amino acid identity particularly in the calcium coordinating residues.

The ANXC3 core region has also been examined, showing a little or no conserved endonexin fold, type II Ca^2+^-binding site or phosphatidylserine-binding sequences. Based on the above analysis, it has been suggested that ANXC3 may not interact with calcium or membranes in a conventional route (Khalaj et al. 2004a, b).

Loss-of-function experiments using gene deletion or RNAi methodology in mammalians resulted in various phenotypes ranging from normal to embryonic lethal (Brachvogel et al. 2003; Clemen et al. 2003). The results are difficult to interpret, partly due to compensatory function of other annexins as well as difference in genetic background of experimental models (Hannon et al. 2003).

Indeed, the first annexin knock out generated for *anxc1* in *D. discoideum* failed to lead to any change in essential cell functions. However, the introduction of low external Ca^2+^ resulted in defects in growth, motility, and chemotaxis of knock out strain, demonstrating a role for annexin C1 in Ca^2+^ homeostasis (Doring et al. 1991, 1995).

There is abundant documentation on the role of calcium in fungal tip growth (Jackson and Heath 1993; Garrill et al. 1993; Heath and Geitmann 2000). A variety of imaging and measuring methods have repeatedly shown that the growing mycelia have a high concentration of calcium at their tips (Kim et al. 2012). Likewise, several reports have demonstrated the role of calcium in polar growth of pollen tubes, root hairs and fern rhizoids. (Steer 1989; Hepler 2005; Clark et al. 2005; Bushart and Roux 2007). These observations along with the localization of annexins at the tip of latter cells can emphasize the role of these calcium binding proteins in growth physiology of plants (Konopka-Postupolska et al. 2011).

To investigate the possible role of fungal annexins in tip growth and protein secretion, an *anxc1* knock out strain of *A. niger* has been generated (Khalaj et al. 2004a). Normal growth phenotype and intact secretion capacity of the KO strain, even in low Ca^2+^ concentrations, suggest a non-essential function of this gene in growth and protein secretion. Disruption of other member of fungal annexins, *anxc3*, in human pathogen *A. fumigatus* resulted in no obvious phenotype related to the growth or protein secretion. A comparative protein profiling of δ*anxc3* mutant revealed the modification of respiratory chain proteins and stress response proteins in this mutant. In this sense, a possible anti-stress function has been proposed for ANXC3 in *A. fumigatus* (Khalaj et al. 2011). In this line, several studies have highlighted the protective role of annexins against various stresses such as oxidative stress, heavy metal stress and osmotic stress in plants (Jami et al. 2008; Konopka-Postupolska et al. 2009; Laohavisit et al. 2010).

The study of annexin-interacting proteins has been an approach in elucidation of the annexin biological functions. As an example, co-purification of a 34-kD annexin protein with (1→3)-β-d-glucan synthase in *Saprolegnia* suggests its role in modulation of this cell wall synthesis enzyme. Add-back experiments using purified annexin have confirmed a positive regulatory effect on glucan synthase activity which needs an optimal ratio of enzyme/annexin (Bouzenzana et al. 2006). Similarly, the presence of two cell-wall associated annexins in the plant pathogen *P. infestans* may indicate the importance of these proteins in mycelia growth and penetration of this pathogen into the host tissue (Grenville-Briggs et al. 2010).

Shirakawa et al. (2005) have identified two aminoacyl-tRNA synthases as annexin binding proteins in true slime mold, *Physarum polycephalum*. They have suggested that there is a possible relationship between membrane trafficking and protein machinery. More studies are needed to confirm such activities in fungi.

## Conclusion

Several potential functions have been assigned to the fungal annexins. The presence of this family in fungi may suggest a role in providing the particular needs of these organisms. The advantage of low numbers of fungal annexins (e.g., 3 in ascomycetes), together with fewer cell types, provides an excellent opportunity for elucidating the biological functions of these proteins. In this case, a comprehensive multi-gene deletion study along with omics-based investigations can give us a deeper insight into the possible role of annexins in fungal development, the adaptation of fungi to different environments and also, pathogenesis. Furthermore, the identification of annexin binding partners and characterization of their possible functional complexes may be helpful in understanding the relevant biological processes.
